# Long-Term Effect of SARS-CoV-2 Infection on the Retinal and Choroidal Microvasculature

**DOI:** 10.3390/jcm12072528

**Published:** 2023-03-27

**Authors:** Magdalena Kal, Mateusz Winiarczyk, Dorota Zarębska-Michaluk, Dominik Odrobina, Elżbieta Cieśla, Bernadetta Płatkowska-Adamska, Michał Biskup, Paweł Pabjan, Stanisław Głuszek, Jerzy Mackiewicz

**Affiliations:** 1 Collegium Medicum, Jan Kochanowski University of Kielce, 25-369 Kielce, Poland; 2 Ophthalmic Clinic, The Voivodeship Hospital, 25-736 Kielce, Poland; 3 Department of Vitreoretinal Surgery, Medical University of Lublin, 20-059 Lublin, Poland; 4 Department of Infectious Disease, Provincial Hospital in Kielce, 25-736 Kielce, Poland; 5 Institute of Medical Science, Jan Kochanowski University of Kielce, 25-369 Kielce, Poland

**Keywords:** COVID-19, SARS-CoV-2, optical coherence tomography angiography, vessel density, persistent microvascular changes

## Abstract

The purpose of this study was to evaluate the persistent changes in microvascular parameters based on optical coherence tomography angiography (OCTA) in patients hospitalized due to COVID-19 bilateral pneumonia. The case-control prospective study was carried out among 49 patients with COVID-19 and 45 healthy age- and gender-matched 2 and 8 months after hospital discharge. We found a significantly decreased vessel density (VD) in superficial capillary plexus (SCP) in COVID-19 patients. Significantly decreased vessel density (VD) in the superficial capillary plexus (SCP), the deep capillary plexus (DCP), and choriocapillaris (CC), with significantly increased vessel density observed in the choriocapillaris in the foveal area (FCC). The foveal avascular zone in DCP (FAZd) was significantly increased in the COVID-19 group. We found differences between OCTA parameters according to gender. The foveal VD in SCP and DCP was significantly decreased in women compared to men. The FAZ area in SCP (FAZs) and superior VD in the choriocapillaris (SCC) were significantly increased in women. In conclusion, we noticed persistent changes in the ocular parameters of OCTA in COVID-19 patients. At the second follow-up visit, we observed a widened FAZ zone in SCP and decreased VD in some regions of the retina and choroid.

## 1. Introduction

The severe acute respiratory syndrome coronavirus 2 (SARS-CoV-2) spread worldwide in December 2019, causing the pandemic announced by World Health Organization (WHO) on 11 March 2020. This virus can cause a life-threatening infection associated with acute respiratory distress syndrome, a prothrombotic condition, cytokine storm, and multiorgan failure [1]. The SARS-CoV-2 virus binds via protein S to the ACE2 receptor present at high rates in the heart, lung, kidney, oleum, bladder epithelia Wells, and gastrointestinal system. This receptor is also present in the retina and choroid of the eye [2]. By damaging endothelial cells, this virus can lead to ischaemia, oedema, and hypercoagulability [3].

In our previous study, we used optical coherence tomography (OCT) to evaluate the retinal and choroidal microvasculature and neuroretinal structures in hospitalized patients with bilateral pneumonia due to SARS-CoV-2. We found a decreased vascular density (VD), enlarged foveal avascular zone (FAZ), thickened retinal nerve fibre layer (RNFL), and thinner ganglion cell layer (GCL) in foveal and parafoveal areas in COVID-19 patients compared to a cohort of age and sex-matched healthy controls [4]. Our results were consistent with those of other authors [5,6]. In the current study, we want to report the results of OCTA 6 months after the first study, in which persistent changes in some of its parameters are present. We decided to separate COVID-19 patients into two groups differentiating on gender due to the differences described in the literature in the course of the viral disease itself, the higher number of hospitalizations, and mortality among men due to COVID-19 [7]. The persisting symptoms such as fatigue and dyspnea, impaired pulmonary function [8,9], and chest image abnormalities due to SARS-CoV-2 infection were described [10]. These symptoms were more common in women than men, although there are no reports more than six months after follow-up [11,12,13].

## 2. Materials and Methods

### 2.1. Subjects

There was a double prospective study, controls in COVID-19 patients with bilateral pneumonia, who were hospitalized in the Department of Infectious Diseases of WSZ in Kielce during the pandemic spring wave caused by B.1.1.7 variant of SARS-CoV-2 from March to May 2021. The project was approved by the Bioethics Committee of Collegium Medicum of Jan Kochanowski University in Kielce (study code 54 approved on 1 July 2021).

As described in our previous paper, we excluded patients with eye and systemic diseases that could have affected the measurement outcomes, as previously described [4].

First, we examined 63 COVID-19 patients with bilateral pneumonia (120 eyes), 2 months after hospital discharge. Among this group we excluded 6 eyes: 2 eyes with hyperopia > 3 diopters, 1 eye with myopia > 3 diopters, 2 eyes after ocular trauma, and 1 eye after uveitis. After 6 months, we contacted these 62 patients and invited them to repeat the ocular examination; 49 patients (75 eyes) agreed to return. Among this group, we excluded the patients with these conditions and incomplete test results.

All patients signed the written informed consent to participate in the current study and underwent ophthalmological evaluation two and eight months after hospital discharge.

### 2.2. Characteristics of the Studied Group

The analyzed COVID-19 group consisted of 49 patients. In all, 31 men (63.27%) and 18 women (36.73%) with COVID-19 bilateral pneumonia participated in the analysis. A total of 75 eyes were included in the study group. The mean age of participants was 51.33 ± 1.45. The mean age of women was 55.1 ± 2.69, and men’s was 49.6 ± 1.66 (*p* = 0.075). The mean (M) ± standard error of BMI M ± SEM was 28.56 (0.50) kg/m^2^. There was no statistically significant difference in BMI between COVID-19 patients and the control group (*p* = 0.051).

The clinical diagnosis of COVID-19 was confirmed as previously described [4].

A total of 49 COVID-19 patients (75 eyes) underwent complete ophthalmic examination 8 months after hospital discharge. The visual acuity and reading vision were measured with a LogMAR scale. The intraocular pressure (IOP) measurement, a slit-lamp examination, OCT of the macula and optic nerve, and angio-OCT (OCTA) were performed. At the follow-up examination, the patients did not report any impairment of near or distant visual acuity or any visual complaints.

### 2.3. Characteristics of a Healthy Group

The control group included healthy patients who attended the ophthalmology department for a routine eye examination. This group consisted of 43 subjects (*n* = 43; eyes = 83) with a mean age of 47.76 ± 1.38. Written informed consent was obtained from all patients. Inclusion criteria for this group were as follows: age of 30–70 years, negative laboratory tests for SARS-CoV-2 infection (PCR from a nasopharyngeal swab), the absence of COVID-19 symptoms in the past or close contact with COVID-19 patients within the 14 days before the examination, and the absence of concomitant eye diseases. All patients had full distance and near vision of 6/6 in Snellen charts (0.0); the spherical equivalent was −0.67 (0.13), and the mean axial length was 23.35 (0.10) mm (Table 1).

### 2.4. Optical Coherence Angiography Measurements

All scans were acquired with Swept Source DRI-OCT Triton SS-OCT Angio (Topcon Inc., Tokyo, Japan). OCTA protocols included the following parameters: 4.5 × 4.5 mm and 6 × 6 mm scanning protocols.

OCTA parameters evaluated were vessel density (VD) in the three different plexi: (superficial capillary plexus) SCP, deep capillary plexus (DCP), and choriocapillaris (CC) using the ETDRS grid subfields to define the areas of interest. The vessel density was measured in superior (S), inner nasal (N), inner inferior (I), and inner temporal (T) ETDRS subfields centered on the macula by fixation. Vessel density measurement was performed via the integrated software. The foveal avascular zone (FAZ) in the SCP and in the DCP was manually delineated by two independent graders, encompassing the central fovea, where no clear and demarcated vessels were seen on the OCTA. Scan quality over 65% was a threshold for eligibility for the study, as previously described [4].

### 2.5. Statistical Analysis

For quantitative traits, distributions were checked. The mean and standard error of the mean (SEM), median (Me), and interquartile range (IQR) were calculated separately for patients tested at 0 months, 6 months, and the control group separately. Student’s t-tests for dependent samples and Wilcoxon’s paired rank test were used to compare the variables for binary studies. For comparison of COVID-19 men and women in the 2 separate studies, and COVID-19 patients studied at months 0 and 6 versus the control group, the Student’s t-test was used for independent samples and the Mann-Whitney, depending on their distribution. A statistical significance threshold of *p* < 0.05 was set. The analysis was performed with STATISTICA 13.3 statistical package, Polish version (STATSOFT, Krakow, Poland).

## 3. Results

### 3.1. OCT Angiography Outcomes

#### 3.1.1. Case-Control Study (6 Months)

In the optical coherence tomography angiography (OCTA) analysis, a significantly decreased vessel density (VD) in superficial capillary plexus (SCP) was observed in COVID-19 patients at the 6-month examination compared to controls in the superior area (S SCP) (45.38 ± 0.40 vs. 48.23 ± 0.37, *p* = 0.000), nasal area (N SCP) (43.91 ± 0.27 vs. 44.99 ± 0.27, *p* = 0.024), inferior area (I SCP) (44.43 ± 0.40 vs. 47.30 ± 0.36, *p* < 0.001), temporal area (T SCP) (44.82 ± 0.23 vs. 46.44 ± 0.24, *p* < 0.001). The foveal avascular zone in SCP (FAZ s) was significantly increased in COVID-19 patients at the 6-month examination, compared to controls (328.95 ± 13.35 vs. 251.03 ± 12.10, *p* < 0.001).

The significantly decreased vessel density (VD) was also found in deep capillary plexus (DCP) in COVID-19 patients at the 6-month examination, compared to controls in foveal area (F DCP) (14.48 ± 0.55 vs. 16.93 ± 0.49, *p* < 0.001), superior area (S DCP) (47.31 ± 0.37 vs. 52.18 ± 0.40, *p* < 0.001), nasal area (N SCP) (45.62 ± 0.31 vs. 48.45 ± 0.32, *p* < 0.001), inferior area (I DCP) (45.83 ± 0.38 vs. 50.91 ± 0.40, *p* < 0.001), temporal area (T DCP) (43.68 ± 0.28 vs. 47.20 ± 0.33, *p* < 0.001). The foveal avascular zone in DCP (FAZd) was significantly increased in COVID-19 patients at the 6-month examination, compared to controls (552.80 ± 23.74 vs. 235.05 ± 12.10, *p* < 0.001).

The significantly decreased vessel density (VD) was observed in the choriocapillaris (CC) in COVID-19 patients at the 6-month examination, compared to controls in the superior area (S CC) (52.07 ± 0.26 vs. 54.24 ± 0.28, *p* < 0.001), inferior area (I CC) (52.24 ± 0.25 vs. 54.26 ± 0.28, *p* < 0.001), temporal area (T CC) (52.99 ± 0.27 vs. 53.50 ± 0.50, *p* < 0.001) (Table 2).

#### 3.1.2. Prospective Cohort Study (0–6 Months)

In the optical coherence tomography angiography (OCTA) analysis, a significantly decreased vessel density (VD) in superficial capillary plexus (SCP) was observed in COVID-19 patients at the 6-month examination, compared to 0 month in the foveal area (F SCP) (19.93 ± 0.48 vs. 20.54 ± 0.51, *p* = 0.006), superior area (S SCP) (45.38 ± 0.40 vs. 48.44 ± 0.31, *p* < 0.001), nasal area (N SCP) (43.91 ± 0.27 vs. 44.92 ± 0.29, *p* = 0.004), inferior area (I SCP) (44.43 ± 0.40 vs. 47.29 ± 0.42, *p* < 0.001).

The significantly decreased vessel density (VD) was observed in deep capillary plexus (DCP) in COVID-19 patients at the 6-month examination, compared to 0 months in the foveal area (F DCP) (14.48 ± 0.55 vs. 17.11 ± 0.55, *p* < 0.001), superior area (S DCP) (47.31 ± 0.37 vs. 52.00 ± 0.35, *p* < 0.001), nasal area (N DCP) (45.62 ± 0.31 vs. 49.05 ± 0.34, *p* < 0.001), inferior area (I DCP) (45.83 ± 0.38 vs. 51.10 ± 0.48, *p* < 0.001).

The foveal avascular zone in DCP (FAZ d) was significantly increased in COVID-19 patients at the 6-month examination, compared to 0 months (552.80 ± 23.74 vs. 359.32 ± 16.92, *p* < 0.001).

The significantly decreased vessel density (VD) was observed in the choriocapillaris (CC) in COVID-19 patients at the 6-month examination, compared to 0 months in the superior area (S CC) (52.07 ± 54.07 ± 0.24, *p* < 0.001), nasal area (N CC) (52.79 ± 0.25 vs. 53.76 ± 0.26, *p* = 0.003), inferior area (I CC) (52.24 ± 0.25 vs. 54.13 ± 0.26, *p* < 0.001), temporal area (T CC) (52.99 ± 0.27 vs. 54.09 ± 0.20, *p* < 0.001).

The significantly increased vessel density (VD) was observed in the choriocapillaris (CC) in COVID-19 patients at the 6-month examination, compared to 0 months in the foveal area (FCC) (52.43 ± 0.34 vs. 51.34 ± 0.47, *p* = 0.014) (Table 3).

### 3.2. Gender Analysis

#### 3.2.1. Gender Analysis at 0 Month

In OCTA analysis, the foveal VD in SCP was significantly decreased in women than in men (18.91 ± 0.85 vs. 21.62 ± 0.58, *p* = 0.008) at 0 months. The VD was significantly decreased in the temporal area in DCP in women than in men (46.55 ± 0.52 vs. 48.04 ± 0.36, *p* = 0.018).

The FAZ area in SCP (FAZs) was significantly increased in women than in men (367.86 ± 22.56 vs. 300.76 ± 13.53, *p* = 0.008) at 0 months (Table 4).

#### 3.2.2. Gender Analysis at the 6th Month

In OCTA analysis, the foveal VD in SCP was significantly decreased in women than in men (18.69 ± 0.88 vs. 20.75 ± 0.53, *p* = 0.037) at the 6th month. The VD was significantly decreased in the temporal area in DCP in women than in men (43.01 ± 0.46 vs. 44.13 ± 0.34, *p* = 0.049).

In OCTA analysis, the superior VD in CC (S CC) was significantly increased in women than in men (52.76 ± 0.45 vs. 51.61 ± 0.29, *p* = 0.028) at the 6th month.

The FAZ area in SCP (FAZs) was significantly increased in women than in men (379.17 ± 23.56 vs. 295.46 ± 13.86, *p* = 0.002) during the 6th month (Table 5).

## 4. Discussion

The current study aimed to evaluate and compare the short- and long-term changes in microvascular parameters based on optical coherence tomography angiography (OCTA) in COVID-19 patients hospitalized due to bilateral pneumonia caused by SARS-CoV-2.

The OCT is a non-invasive tool, which can successfully visualize the microvascular network of the retina and choroid in many ocular disorders and general diseases [14].

The results of our study were compared with the results of other researchers who evaluated the condition of the retina and choroid six months after the first examination, and, like them, we assessed whether the changes in the eye have a more severe course in women compared with men [15,16].

In our first study, we found decreased vessel density (VD) in the superficial capillary plexus (SCP), in the deep capillary plexus (DCP), and in the choriocapillaris (CC) in 63 COVID-19 patients, compared to a cohort of age and sex-matched healthy controls. The foveal avascular zone (FAZ) in SCP and in DCP were significantly increased in that study [4].

Six months later, we examined 49 of 63 hospitalized COVID-19 patients with bilateral pneumonia 8 months before and found a significantly decreased vessel density (VD) in superficial capillary plexus (SCP) in OCTA in COVID-19 patients at the 6-month examination, compared to controls in parafoveal areas and at the 6-month examination, compared to 0 months in foveal and parafoveal areas.

The foveal avascular zone in SCP (FAZ s) was significantly increased in COVID-19 patients at the 6-month examination, compared to controls.

We observed a significantly decreased vessel density (VD) in deep capillary plexus (DCP) in COVID-19 patients at the 6-month examination, compared to controls and compared to 0 months in foveal and parafoveal areas.

The foveal avascular zone in DCP (FAZ d) was significantly increased in COVID-19 patients at the 6-month examination, compared to controls and compared to 0 month.

The significantly decreased vessel density (VD) was observed in choriocapillaris (CC) in COVID-19 patients at the 6-month examination, compared to controls in parafoveal areas.

We found a significantly decreased vessel density (VD) in choriocapillaris (CC) in parafoveal areas and a significantly increased vessel density (VD) in foveal area of choriocapillaris (FCC) in COVID-19 patients at 6-month examination, compared to 0 month.

Changes in OCTA microvascular parameters described two months after hospitalization for SARS-CoV-2 infection are still present six months later. The similar findings may involve tissues that are embryologically and structurally similar to the retina, such as the brain. One study reports microstructural abnormalities and changes in cerebral blood flow three months after recovery from pneumonia in COVID-19 patients [17].

Many papers report persistent changes after SARS-CoV-2 infection, such as pulmonary dysfunction six months after the onset of symptoms, renal dysfunction, vascular dysfunction, and thromboembolic disease even one month after the illness [18,19].

SARS-CoV-2 can directly destroy endothelial cells by binding to angiotensin-converting enzyme 2 (ACE-2) [20,21]. This mechanism can develop coagulopathy leading to damage to the vessels in many human organs [22]. The retina and choroid are richly vascularized structures with the ACE2 receptor present, which is a choke point for the SARS-CoV-2 virus, so one might expect microangiopathy here. Many authors have described thrombotic-related findings in the retina, i.e., retinal haemorrhages, cotton wool balls, and dilated and tortuous retinal vessels in COVID-19 patients [23,24].

We compared parameters of OCTA between women and men at 0 months and at 6 months. Many studies have described persisting symptoms as more common in women, such as fatigue and dyspnea, impaired pulmonary function [8], and chest image abnormalities [10,13]. One may hypothesize that COVID-19 infection may lead to different sex-related complications.

The foveal VD in SCP was significantly decreased in women than in men at 0 months and 6 months. The VD was also significantly decreased in the temporal area in DCP in women than in men at 0 months and 6 months. The FAZ area in SCP (FAZs) was significantly increased in women than in men at 0 months and 6 months. The superior VD in the choriocapillaris (SCC) was significantly increased in women than in men at six months.

Some authors suggest a slower recovery process in women compared to men or even a gradual deterioration of ocular parameters based on the OCTA study in this group. This may suggest that women may have a higher risk of reduced VD in the months following COVID-19. Some researchers also report that women may be more predisposed to persistent neurological and psychological impairment, fatigue, post-activity polypnea, and alopecia [13,25].

Fernandez-de-las Penas C. et al. described that the number of post-COVID symptoms was significantly higher in women than in men (*p* < 0.001). Furthermore, he found that women were more predisposed to post-COVID symptomatology even eight months after hospital discharge [26]. Three multicenter studies reported that the female gender is a potential risk factor for the development of post-COVID symptoms, e.g., fatigue, dyspnea, or dermatological symptoms [27].

The issue of why the female sex is more predisposed to post-COVID symptoms is currently debated in the literature. First, there are biological differences in the expression of ACE2 I transmembrane protease serine 2 (TMPRSS2) receptors between males and females, and immunological differences; lower production of pro-inflammatory interleukin-6 (IL-6) due to viral infection in women [28,29]. Other factors that are considered in the more frequent development of post-COVID symptoms in women are more frequent hand washing, less exposure to infections, and higher psychological stress [30].

Many researchers have considered the effect of female hormones on the vascularization of the eyeball. Estrogen, progesterone, and testosterone are regulators of blood flow through the retina and choroid and are also key to regulating vascular tone in the body. Estrogen plays a protective role when there is a decrease in vascular resistance in the large vessels of the eye [28,29]. One study showed significantly greater choroidal blood flow in women under 40 compared to women over 55 men. Age was not significant for choroidal blood flow in men. Also, pulsatile ocular blood flow and pulse amplitude were significantly higher in pre-menopausal women compared to age-matched male and post-menopausal women not taking hormonal therapy [30]. Further sex-disaggregated studies evaluating retinal and choroidal blood circulation are needed.

Our study had several limitations. The separate groups of women and men consisted of too few individuals. An algorithm for the OCT examination is needed to assess which parameters of this examination may be highly helpful in assessing the ocular condition and general state of COVID-19 patients. The strength of our study was the highly selected group of patients hospitalized for SARS-CoV-2 infection and the strictly maintained time frame of the two follow-up examinations in this group.

## 5. Conclusions

A study that we performed twice in COVID-19 patients, two and six months after hospital discharge, showed a trend toward persistent decline in VD in retinal and choroidal vascular plexuses. Differences were observed between OCTA parameters between women and men to the disadvantage of the first group, such as reduced VD in SCP and in DCP and a widened FAZ zone in SCP. Further follow-up studies are needed in this group of patients stratified by sex to assess whether there are consequences in these ocular structures due to poor blood supply.

## Figures and Tables

**Table 1:** Ocular characteristics of COVID-19 patients at 0 months and 6 months.

Parameters	0 Month		6 Months		*p*
	$\bar{x}$ (SEM)	Me (IQR)	$\bar{x}$ (SEM)	Me (IQR)	
Visual acuity	6/6 (0.0)	6/6 (0.0)	6/6 (0.0)	6/6 (0.0)	0.593 ^B^
Reading vision	6/6 (0.0)	6/6 (0.0)	6/6 (0.0)	6/6 (0.0)	0.285 ^B^
Axial length	23.56 (0.10)	23.67 (1.1)	23.56 (0.10)	23.67 (1.1)	-
Intraocular pressure in mmHg (IOP)	16.66 (0.31)	16.85(4.05)	16.42 (0.31)	16.40 (3.70)	0.504 ^A^
Spherical equivalent (D)	0.19 (0.16)	0.0 (2.38)	0.20 (0.16)	0.0 (2.25)	0.673 ^A^

^A^—t Student test; ^B^—Wilcoxon rank test; D—diopters.

**Table 2:** Comparison of foveal (F) and parafoveal parameters of OCTA (optical coherence tomography angiography) between COVID-19 patients (women and men) in the 6-month and control group: SCP—superficial capillary plexus, DCP—deep capillary plexus, CC—choriocapillaris, FAZ s—superficial foveal avascular zone, FAZ d—deep foveal avascular zone, F—foveal area, S—superior area, N—nasal area, I—inferior area, T—temporal area. Mean ± SEM (standard error of the mean) structural OCT values. Bold values denote statistical significance at the *p* < 0.05 level.

Variables	6th Month (*n* = 75 Eyes)		Control Group (*n* = 83 Eyes)		*p*
	$\bar{x}$ (SEM)	Me (IQR)	$\bar{x}$ (SEM)	Me (IQR)	
**F SCP**	19.93 (0.48)	19.86 (5.30)	20.70 (045)	21.14 (5.34)	0.239 ^A^
**F DCP**	14.48 (0.55)	13.89 (6.11)	16.93 (0.49)	16.55 (6.92)	<0.001 ^A^
**F CCP**	52.43 (0.34)	52.16 (2.97)	52.43 (0.47)	52.96 (6.38)	0.404 ^B^
**S SCP**	45.38 (0.40)	46.26 (3.65)	48.23 (0.37)	48.19 (3.84)	0.000 ^B^
**S DCP**	47.31 (0.37)	46.88 (3.41)	52.18 (0.40)	51.89 (4.63)	<0.001 ^B^
**S CCP**	52.07 (0.26)	52.19 (3.59)	54.24 (0.28)	54.23 (2.44)	<0.001 ^B^
**N SCP**	43.91 (0.27)	44.59 (2.70)	44.99 (0.27)	45.05 (3.57)	0.024 ^B^
**N DCP**	45.62 (0.31)	45.60 (4.41)	48.45 (0.32)	47.91 (4.18)	<0.001 ^A^
**N CCP**	52.79 (0.25)	52.75 (2.85)	53.22 (0.25)	53.32 (2.89)	0.148 ^B^
**I SCP**	44.43 (0.40)	45.22 (5.06)	47.30 (0.36)	47.45 (5.13)	<0.001 ^B^
**I DCP**	45.83 (0.38)	45.06 (4.02)	50.91 (0.40)	50.34 (4.39)	<0.001 ^B^
**I CCP**	52.24 (0.25)	52.19 (3.10)	54.26 (0.28)	54.11 (2.45)	<0.001 ^B^
**T SCP**	44.82 (0.23)	44.77 (2.06)	46.44 (0.24)	46.52 (3.29)	<0.001 ^A^
**T DCP**	43.68 (0.28)	43.36 (3.30)	47.20 (0.33)	46.93 (4.39)	<0.001 ^A^
**T CCP**	52.99 (0.27)	53.00 (2.65)	53.50 (0.50)	54.06 (2.43)	<0.001 ^B^
**FAZs (µm^2^)**	328.95 (13.35)	329.59 (128.40)	251.03 (12.10)	243.07 (154.03)	<0.001 ^A^
**FAZd (µm^2^)**	552.80 (23.74)	562.23 (325.06)	235.05 (12.10)	226.41 (157.03)	<0.001 ^B^

^A^—t Student test; ^B^—Wilcoxon rank test.

**Table 3:** Comparison of foveal (F) and parafoveal parameters of OCTA (optical coherence tomography angiography) between 0 months and the 6th month in all COVID-19 patients (women and men): SCP—superficial capillary plexus, DCP—deep capillary plexus, CC—choriocapillaris, FAZ s—superficial foveal avascular zone, FAZ d—deep foveal avascular zone, F—foveal area, S—superior area, N—nasal area, I—inferior area, T—temporal area. Mean ± SEM (standard error of the mean) structural OCT values. Bold values denote statistical significance at the *p* < 0.05 level.

Variables OCTA	0 Month (*n* = 75 Eyes)		6th Month (*n* = 75 Eyes)		*p*
	$\bar{x}$ (SEM)	Me (IQR)	$\bar{x}$ (SEM)	Me (IQR)	
**F SCP**	20.54 (0.51)	20.79 (4.97)	19.93 (0.48)	19.86 (5.30)	0.006 ^A^
**F DCP**	17.11 (0.55)	16.92 (5.75)	14.48 (0.55)	13.89 (6.11)	<0.001 ^A^
**F CC**	51.34 (0.47)	51.64 (4.91)	52.43 (0.34)	52.16 (2.97)	0.014 ^B^
**S SCP**	48.44 (0.31)	48.58 (2.75)	45.38 (0.40)	46.26 (3.65)	<0.001 ^B^
**S DCP**	52.00 (0.35)	52.04 (4.61)	47.31 (0.37)	46.88 (3.41)	<0.001 ^A^
**S CC**	54.07 (0.24)	54.12 (2.43)	52.07 (0.26)	52.19 (3.59)	<0.001 ^B^
**N SCP**	44.92 (0.29)	45.13 (3.95)	43.91 (0.27)	44.59 (2.70)	0.004 ^A^
**N DCP**	49.05 (0.34)	48.51 (3.64)	45.62 (0.31)	45.60 (4.41)	<0.001 ^B^
**N CC**	53.76 (0.26)	53.83 (2.67)	52.79 (0.25)	52.75 (2.85)	0.003 ^A^
**I SCP**	47.29 (0.42)	48.01 (3.86)	44.43 (0.40)	45.22 (5.06)	<0.001 ^B^
**I DCP**	51.10 (0.48)	51.14 (5.19)	45.83 (0.38)	45.06 (4.02)	<0.001 ^B^
**I CC**	54.13 (0.26)	54.25 (3.37)	52.24 (0.25)	52.19 (3.10)	<0.001 ^A^
**T SCP**	46.57 (0.24)	46.45 (3.16)	44.82 (0.23)	44.77 (2.06)	<0.001 ^A^
**T DCP**	47.44 (0.31)	47.59 (3.88)	43.68 (0.28)	43.36 (3.30)	<0.001 ^A^
**T CCP**	54.09 (0.20)	53.94 (2.40)	52.99 (0.27)	53.00 (2.65)	<0.001 ^A^
**FAZs (µm^2^)**	327.60 (12.64)	323.55 (128.40)	328.95 (13.35)	329.59 (128.40)	0.822 ^A^
**FAZd (µm^2^)**	359.32 (16.29)	343.11 (155.06)	552.80 (23.74)	562.23 (325.06)	<0.001 ^B^

^A^—t Student test; ^B^—Wilcoxon rank test.

**Table 4:** Comparison of foveal (F) and parafoveal parameters of OCTA (optical coherence tomography angiography) parameters between women and men in COVID-19 patients in 0 month: SCP-superficial capillary plexus, DCP—deep capillary plexus, CC—choriocapillaris, FAZ s—superficial foveal avascular zone, FAZ d—deep foveal avascular zone, F—foveal area, S—superior area, N—nasal area, I—inferior area, T—temporal area. Mean ± SEM (standard error of the mean) structural OCT values. Bold values denote statistical significance at the *p* < 0.05 level.

Variables	Women—0 Month (*n* = 30 Eyes)		Men—0 Month (*n* = 45 Eyes)		*p*
	$\bar{x}$ (SEM)	Me (IQR)	$\bar{x}$ (SEM)	Me (IQR)	
**F SCP**	18.91 (0.85)	18.89 (3.75)	21.62 (0.58)	21.50 (4.04)	0.008 ^A^
**F DCP**	16.96 (0.83)	17.74 (5.00)	17.21 (0.74)	16.33 (5.65)	0.693 ^B^
**F CC**	50.62 (0.83)	50.78 (5.48)	51.83 (0.55)	52.56 (3.74)	0.143 ^B^
**S SCP**	49.11 (0.44)	49.41 (2.48)	47.99 (0.41)	48.44 (2.89)	0.071 ^B^
**S DCP**	51.44 (0.66)	51.66 (5.27)	52.37 (0.39)	52.45 (4.00)	0.178 ^A^
**S CC**	54.61 (0.44)	54.49 (2.19)	53.71 (0.26)	53.71 (1.84)	0.097 ^B^
**N SCP**	45.38 (0.39)	45.79 (3.59)	44.62 (0.41)	44.70 (4.40)	0.221 ^B^
**N DCP**	48.66 (0.62)	47.56 (4.43)	49.31 (0.39)	48.82 (2.68)	0.077 ^B^
**N CC**	53.81 (0.38)	53.63 (2.17)	53.73 (0.35)	54.26 (3.00)	0.868 ^A^
**I SCP**	48.31 (0.44)	48.46 (2.54)	46.61 (0.61)	47.62 (4.77)	0.083 ^B^
**I DCP**	50.42 (0.68)	49.98 (3.18)	51.55 (0.66)	51.74 (5.23)	0.089 ^B^
**I CC**	54.25 (0.39)	54.29 (3.73)	54.04 (0.35)	54.05 (3.05)	0.727 ^A^
**T SCP**	46.93 (0.35)	46.64 (2.54)	46.33 (0.36)	46.22 (3.28)	0.258 ^A^
**T DCP**	46.55 (0.52)	46.38 (3.68)	48.04 (0.36)	47.95 (3.46)	0.018 ^A^
**T CC**	54.14 (0.32)	53.81 (2.33)	54.06 (0.25)	53.98 (1.97)	0.847 ^A^
**FAZs (µm^2^)**	367.86 (22.56)	367.77 (114.43)	300.76 (13.53)	297.32 (101.35)	0.008 ^A^
**FAZd (µm^2^)**	366.22 (24.63)	346.34 (115.90)	349.73 (21.80)	326.98 (161.64)	0.398 ^B^

^A^—t Student test; ^B^—Wilcoxon rank test.

**Table 5:** Comparison of foveal (F) and parafoveal parameters of OCTA (optical coherence tomography angiography) parameters between women and men in the 6th month in COVID-19 patients: SCP—superficial capillary plexus, DCP—deep capillary plexus, CC—choriocapillaris, FAZ s—superficial foveal avascular zone, FAZ d—deep foveal avascular zone, F—foveal area, S—superior area, N—nasal area, I—inferior area, T—temporal area. Mean ± SEM (standard error of the mean) structural OCT values. Bold values denote statistical significance at the *p* < 0.05 level.

Variables	Women—6th Month (*n* = 30 Eyes)		Men—6th Month (*n* = 45 Eyes)		*p*
	$\bar{x}$ (SEM)	Me (IQR)	$\bar{x}$ (SEM)	Me (IQR)	
**F SCP**	18.69 (0.88)	18.96 (5.29)	20.75 (0.53)	20.56 (4.46)	0.037 ^A^
**F DCP**	14.23 (0.92)	14.15 (6.25)	14.64 (0.70)	13.71 (5.61)	0.876 ^B^
**F CCP**	51.91 (0.53)	51.69 (3.01)	52.78 (0.43)	52.55 (2.91)	0.209 ^A^
**S SCP**	45.66 (0.63)	46.50 (3.62)	45.19 (0.52)	45.89 (3.49)	0.632 ^B^
**S DCP**	47.31 (0.62)	47.26 (4.55)	47.31 (0.46)	46.39 (3.09)	0.885 ^B^
**S CCP**	52.76 (0.45)	53.74 (3.45)	51.61 (0.29)	51.69 (2.40)	0.028 ^A^
**N SCP**	44.50 (0.37)	44.87 (2.29)	43.51 (0.36)	43.87 (2.89)	0.055 ^B^
**N DCP**	46.22 (0.42)	46.30 (3.50)	45.21 (0.43)	45.23 (4.56)	0.112 ^A^
**N CCP**	52.59 (0.35)	52.55 (1.82)	52.92 (0.34)	53.30 (3.01)	0.523 ^A^
**I SCP**	44.45 (0.59)	44.74 (3.51)	44.42 (0.54)	45.51 (5.35)	0.751 ^B^
**I DCP**	45.46 (45.46)	44.72 (2.78)	46.08 (0.50)	45.193.87)	0.274 ^B^
**I CCP**	52.67 (0.40)	52.38 (2.81)	51.95 (0.33)	52.02 (3.46)	0.914 ^A^
**T SCP**	44.82 (0.28)	45.02 (1.72)	44.82 (0.35)	44.75 (2.38)	0.991 ^A^
**T DCP**	43.01 (0.46)	43.26 (3.63)	44.13 (0.34)	43.65 (3.37)	0.049 ^A^
**T CCP**	52.94 (0.38)	52.90 (2.76)	53.03 (0.38)	53.04 (2.75)	0.773 ^B^
**FAZs (µm^2^)**	379.17 (23.56)	381.50 (125.11)	295.46 (13.86)	289.75 (115.91)	0.002 ^A^
**FAZd (µm^2^)**	591.75 (40.67)	581.79 (302.26)	526.84 (28.54)	551.51 (301.16)	0.182 ^A^

^A^—t Student test; ^B^—Wilcoxon rank test.

## Data Availability

Not available.
